# MDDOmics: multi-omics resource of major depressive disorder

**DOI:** 10.1093/database/baae042

**Published:** 2024-06-25

**Authors:** Yichao Zhao, Ju Xiang, Xingyuan Shi, Pengzhen Jia, Yan Zhang, Min Li

**Affiliations:** School of Computer Science and Engineering, Central South University, No.932 South Lushan Road, Changsha 410083, China; School of Computer and Communication Engineering, Changsha University of Science and Technology, No.45 Chiling Road, Changsha 410114, China; School of Computer Science and Engineering, Central South University, No.932 South Lushan Road, Changsha 410083, China; School of Computer Science and Engineering, Central South University, No.932 South Lushan Road, Changsha 410083, China; Department of Psychiatry, and National Clinical Research Center for Mental Disorders, The Second Xiangya Hospital of Central South University, No.139 Renmin Road Central, Changsha 410011, China; School of Computer Science and Engineering, Central South University, No.932 South Lushan Road, Changsha 410083, China

## Abstract

Major depressive disorder (MDD) is a pressing global health issue. Its pathogenesis remains elusive, but numerous studies have revealed its intricate associations with various biological factors. Consequently, there is an urgent need for a comprehensive multi-omics resource to help researchers in conducting multi-omics data analysis for MDD. To address this issue, we constructed the MDDOmics database (Major Depressive Disorder Omics, (https://www.csuligroup.com/MDDOmics/), which integrates an extensive collection of published multi-omics data related to MDD. The database contains 41 222 entries of MDD research results and several original datasets, including Single Nucleotide Polymorphisms, genes, non-coding RNAs, DNA methylations, metabolites and proteins, and offers various interfaces for searching and visualization. We also provide extensive downstream analyses of the collected MDD data, including differential analysis, enrichment analysis and disease-gene prediction. Moreover, the database also incorporates multi-omics data for bipolar disorder, schizophrenia and anxiety disorder, due to the challenge in differentiating MDD from similar psychiatric disorders. In conclusion, by leveraging the rich content and online interfaces from MDDOmics, researchers can conduct more comprehensive analyses of MDD and its similar disorders from various perspectives, thereby gaining a deeper understanding of potential MDD biomarkers and intricate disease pathogenesis.

**Database URL**: https://www.csuligroup.com/MDDOmics/

## Introduction

Major depressive disorder (MDD) is a globally prevalent psychiatric disorder characterized by persistent episodes of depressed mood and/or loss of pleasure in activities for long periods (1). The World Health Organization’s 2023 statistics report states that 3.8% of the global population suffers from MDD, including 5% of adults and 5.7% of adults older than 60 years (2). In addition, MDD is a severely disabling disorder (3, 4). Different from the general population, individuals diagnosed with MDD are more inclined to self-harm, suicidal behaviours and concurrent illnesses, which severely affect their daily lives. Since MDD can manifest in multiple forms with different combinations of heterogeneous symptoms, it remains a challenge for accurate clinical diagnosis and treatment, especially in the context of other illnesses (5, 6). As a result, there is growing attention to this disorder among the public. However, no exact pathological theory can independently account for its pathogenesis (7). An increasing amount of evidence indicates that both genetic and environmental factors contribute to MDD (8, 9), affecting patients at multiple omics levels. For example, genome-wide association studies (GWAS) conducted on diverse populations have identified many SNP loci related to MDD (10, 11). Differential analysis of diverse tissue samples has found specific differentially expressed genes and methylation sites in MDD patients (12–14). Studies on metabolites and proteins in urine and blood also revealed numerous potential biomarkers for MDD (15, 16).

A significant portion of current research on MDD is based on single-omic data (17). Single-omics data analysis is valuable in specific research scenarios, but it has limitations in capturing the intricacy of complex disorders (like MDD, which involves myriad factors) from a singular perspective. Integrative multi-omics data analysis has been proven to be capable of offering a more in-depth and comprehensive solution for diseases (18–20). ProMENDA (21) is a comprehensive repository that integrates all known metabolomic and proteomic data related to MDD. It collected 18 164 differential metabolite entries from 1018 studies, as well as 19 553 differential protein entries from 207 studies. Despite its breadth, ProMENDA does not provide analytical results or visualization interfaces for its collected data, and its scope is confined to the above two types of omics data. Consequently, there is an urgent need to provide a more comprehensive multi-omics resource to assist researchers in their multifaceted analyses for MDD.

In this paper, we have established a database platform named ‘MDDOmics’ (Major Depressive Disorder Omics, https://www.csuligroup.com/MDDOmics/), which integrates 41 222 multi-omics data entries and several original datasets related to MDD, sourced from published studies and public databases and complemented by our analytical results. This comprehensive compilation spans a series of omics data, including SNPs, genes, non-coding RNAs (ncRNAs), DNA methylations, metabolites and proteins. To facilitate a deeper understanding of this data, MDDOmics is equipped with a suite of visualization interfaces, which allow researchers to explore and interpret complex datasets intuitively. We have employed various computational methods to analyse the public data, including differentially expressed gene analysis, differential methylation analysis and enrichment analysis. Moreover, MDDOmics offers prediction results of potential MDD-associated genes and ncRNAs generated using a network impulsive dynamics-based approach. Considering the clinical challenges in distinguishing MDD from similar psychiatric disorders, MDDOmics also collected multi-omics data on schizophrenia, bipolar disorder and anxiety disorder, which may help to discern these intricate disorders with greater precision.

## Materials and methods

### Public data sources

There are three types of public data sources used to extract MDD-related data in this study: the comprehensive databases for disease-related entities (22) (e.g. DisGeNET (23) v7.0, RNADisease v4.0 (24), CircR2Disease v2.0 (25) and LncRNADisease v3.0 (26)), the disease-specific databases (e.g. SZDB v2.0 (27), SZGR v2.0 (28) and dbBIP (29)) and the authoritative literature related to MDD.

Regarding the comprehensive databases, we have extracted most of the related SNP and gene data from DisGeNET v7.0 (23), as well as the related ncRNA data from DisGeNET (23) v7.0, RNADisease v4.0 (24), CircR2Disease v2.0 (25) and LncRNADisease v3.0 (26). In addition, we collected MDD-related expression and methylation data from the NCBI’s Gene Expression Omnibus (GEO) (12, 30–33) database for differential analysis. For the disease-specific databases, we have extracted the MDD-related metabolites and proteins from the ProMENDA (21) database, the schizophrenia multi-omics data from SZDB v2.0 (27) and SZGR v2.0 (28), as well as the bipolar disorder data from dbBIP (29). Data manually retrieved from the literature account for a minor portion of the total multi-omics data, which was obtained by querying PubMed using disease-related keywords and handpicking several essential findings from recent years.

We curated and annotated the collected data with the help of publicly available knowledge. We employed the HUGO Gene Nomenclature Committee (HGNC) (34) database to harmonize the gene symbols. For the annotation of genes and SNPs, we considered such databases as Gene (35) and dbSNP (36), as well as R packages like biomaRt (37) and clusterProfiler (38). As for the ncRNA data, we used the LNCipedia (39) database for lncRNAs, the Circad (40) and circBase (41) databases for circRNAs and the miRBase (42) database for miRNA.

In order to enrich visualization information, we leveraged data from the STRING (43) database and WashU EpiGenome Browser (44). To assist in downstream analyses, we also utilized network data from STRING (43), RNAInter (45), the Biological General Repository for Interaction Datasets (46), HumanNet (47), TissueNet (48) and HumanBase (49), as well as several datasets mentioned in the Weighted Enrichment Analysis Tool (WEAT) (50) method.

In addition to research-related entries, we also collected several original multi-omics datasets related to MDD to facilitate further study by researchers. We collected expression and methylation datasets from the GEO (33) database, GWAS results from organizations like Psychiatric Genomics Consortium (10, 11, 51–53), CONVERGE (54), iPSYCH (55), etc. (56), and metabolite and protein level data from Metabolite Workbench (57) and ProteomeXchange (58), respectively.

### Differential analysis

We retrieved several credible MDD-related methylation and expression datasets from the GEO database, focusing on brain tissue and blood samples. Some datasets were subsequently segmented by tissue (e.g. different brain regions) or batch, in order to maximize the specificity of various tissues and reduce the biases caused by batch effects.

We primarily used the R package ‘ChAMP’ (59) to analyse the methylation data. To minimize inaccuracies introduced by experiments and samples, we did normalization and corrected batch effects on covariates affecting the data. Subsequently, we employed the provided functions to identify the differential methylation sites and regions between control and case groups. For the gene expression datasets, we mainly used the R package ‘limma’ (60) for analysis, which has been a widely recognized tool for gene discovery through differential expression analysis. Similarly, we adjusted the covariates such as age, gender, brain pH value, etc. The results of the analysis were all integrated into the MDDOmics.

### Enrichment analysis

We have conducted the enrichment analysis of the Kyoto Encyclopedia of Genes and Genomes (KEGG) pathway and the Gene Ontology (GO) term on the collected MDD-related genes and subsequently integrated the results into the database. Initially, we adopted the conventional method based on the hypergeometric distribution, which is a prevalent approach for enrichment analysis. This method treats each gene in a set with equal significance. However, in practical scenarios, different genes corresponding to biological pathways and terms often vary in their contributions and importance. This suggests that the classical method may lead to biased results. To address this, we turned to the WEAT (50) method, a weighted functional enrichment method based on the hypergeometric test by introducing a prior weight to each gene. This method prioritizes GO terms or KEGG pathways based on a higher essentiality score from hitting genes rather than the sheer number of hits.

First, we standardized all MDD-related genes collected in our database into the form of Entrez IDs and entered them into the R package clusterProfiler (38) for the conventional enrichment analysis and the WEAT (50) online website for the weighted functional enrichment analysis. Regarding the essentiality score, we used the disease-gene association score from the DisGeNET (23) database, where a higher score for a gene indicates a stronger association with MDD. Additionally, we selected the gene essentiality score derived from expression profiles of the blood and brain tissues in GTEx, which was derived from the WEAT method study (50, 61). This decision is based on the pivotal role of these tissues in MDD research: the brain is the primary site of MDD disorder pathology, while blood serves as a minimally invasive source that can reflect molecular changes related to MDD, such as immune and inflammatory molecules (17, 62–65).

### Disease-gene prediction

In MDDOmics, we have predicted MDD-related candidate genes and ncRNAs by conducting a network propagation process—network impulsive dynamics on a comprehensive biological network, which has been demonstrated to be highly effective in the case study of MDD (66). We constructed the comprehensive network by integrating multiple types of associations, including disease-gene and ncRNA associations from MDDOmics, as well as the protein–protein interactions (PPIs) (43, 46–49), ncRNA-gene (45) associations and so on. We also chose the text-mining-based method (67) to construct a disease similarity network using disease symptoms. Several PPI networks are integrated as options, which include comprehensive and tissue-specific PPI networks. We selected the comprehensive PPI networks from STRING (43), BIOGRID (46) and HumanNet (47) databases, along with the brain tissue-specific networks sourced from the TissueNet (48) and HumanBase (49) databases.

Of particular note, in order to speed up the algorithm, we have applied certain filtering to large networks. When using the ncRNA-ncRNA/gene networks from the RNADisease (24) database, we only used edges with strong interactions. For large networks from HumanBase and STRING, we set thresholds of 0.2 and 0.4 to filter out low-confidence edges. In addition, to construct a heterogeneous network among different types of networks, we uniformly converted all the nodes’ names to gene symbols using the HGNC (34) online tool.

### Visualization

For the collected MDD-related genes, we constructed a comprehensive protein–protein physical interaction network by using data from the STRING (43) database. This network not only enables users to gain insight into the molecular-level interactions of these genes but also assists in identifying potential essential genes through the core nodes, which could be crucial for understanding the biological mechanism underlying MDD.

Additionally, to highlight the similarities and differences between MDD and related disorders, we developed a visualization interface to showcase the cross-analysis of these four psychiatric disorders from the perspectives of both diseases and biological factors.

To clearly visualize the exact chromosomal locations of potential biological factors related to MDD, our platform displays the chromosomal coordinates of all related SNPs, genes, ncRNAs and DNA methylations. This helps researchers swiftly locate their areas of interest, greatly facilitating subsequent experimental planning. Furthermore, we have integrated two tracks from the WashU EpiGenome Browser (44), providing users with more detailed annotation information.

## Results

### Database summary

**Figure 1.**
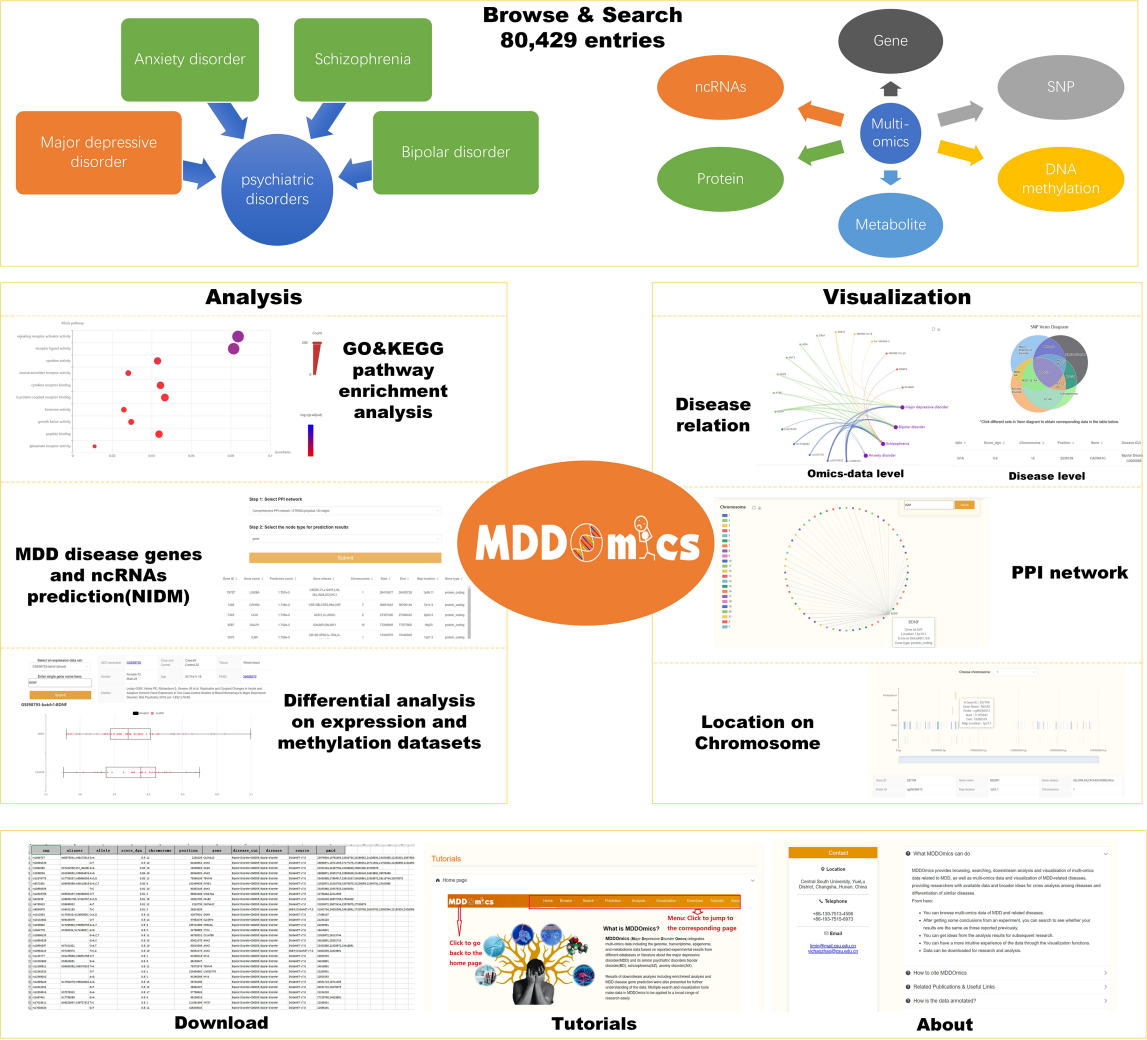
MDDOmics overall framework, which includes seven types of interfaces to the database.

MDDOmics has integrated a total of 80 429 biological entries and several datasets from six omics across four psychiatric disorders, with a primary focus on MDD. Figure 1 displays the framework of the database, detailing the organization of its various pages. The primary data are displayed through the ‘Browse’ and ‘Search’ sections. The ‘Analysis’ section presents a suite of analytical results pertaining to collected MDD data, encompassing GO and KEGG pathway enrichment analyses using both conventional and WEAT (50) methods, as well as differential expression and methylation analyses, and the prediction of MDD-related candidate genes and ncRNAs. The ‘Visualization’ section includes three interfaces: ‘PPI’, ‘Relation’ and ‘Location’, offering interactive graphical representations. Furthermore, users can access data downloads, instructional guides and other database information on the ‘Download’, ‘Tutorial’ and ‘About’ pages, respectively.

### Web implementation

The website overall adopts a front-end and back-end separation framework, utilizing an efficient and flexible technology stack. The front end uses Vue2 (https://v2.vuejs.org/) as the core framework, providing a data-driven view layer that efficiently manages dynamic updates to the user interface. By integrating Axios (https://github.com/axios/axios), the project achieves asynchronous HyperText Transfer Protocol communication with back-end services. It also uses the Element-UI (https://element.eleme.io) framework, which supplies a comprehensive set of pre-defined components. Moreover, it incorporates two powerful visualization toolkits, ECharts (https://echarts.apache.org) and highcharts (https://api.highcharts.com), for constructing highly interactive data visualization charts. The back end adopts the SpringBoot (https://spring.io/projects/spring-boot) framework, using its auto-configuration feature to simplify the configuration work. Moreover, MyBatis (https://blog.mybatis.org) and MyBatis-Plus (https://baomidou.com) are used for data persistence operations. The database system employs MySQL (https://www.mysql.com) as the storage solution, which is responsible for holding all multi-omics data.

The website is freely available to all without the need for registration or login. Designed for cross-platform compatibility, it ensures a user-friendly interface across computers, smartphones and tablets for broad accessibility.

### Data statistics

As shown in Table 1, we collected 41 222 entries about MDD from various public databases and literature. Most SNP and gene data were sourced from the DisGeNET (23) database. The majority of ncRNA entries were from DisGeNET (23) and RNADisease (24) databases. DNA methylation data, limited in number, were exclusively collected from manually curated literature. The relevant protein and metabolite entries were entirely sourced from the ProMENDA (21) database. Besides, we have also collected several publicly available original datasets from studies on human MDD. These include 39 gene expression datasets, 13 ncRNA level datasets, 6 DNA methylation datasets, GWAS results from 4 organizations, 8 protein level datasets and 7 metabolite level datasets. Links to these datasets are provided on the ‘Download’ page for further investigation.

**Table 1.** MDDOmics data statistics

Disease	Omics	Source	Number of entries		
MDD	SNP	DisGeNET v7.0 (76)	1469	1474	41 222
		Manual retrieval	45		
	Gene	DisGeNET v7.0	1209	1396	
		Manual retrieval	245		
	ncRNA	DisGeNET v7.0	59	369	
		RNADisease v4.0 (77)	309		
		CircR2Disease v2.0 (78)	5		
		LncRNADisease v3.0 (79)	5		
	Methylation	Manual retrieval	501	501	
	Metabolite	ProMENDA (80)	18 039	18 039	
	Protein	ProMENDA	19 443	19 443	
Bipolar disorder	SNP	DisGeNET v7.0	874	2944	10 839
		dbBIP (81)	2081		
		Manual retrieval	21		
	Gene	DisGeNET v7.0	1112	6837	
		dbBIP	6246		
	ncRNA	DisGeNET V7.0	48	1058	
		RNADisease v4.0	77		
		dbBIP	939		
		LncRNADisease v3.0	5		
Schizophrenia	SNP	DisGeNET V7.0	2933	3131	27 369
		SZDB2.0 (82)	402		
	Gene	DisGeNET V7.0	2649	11 478	
		SZDB2.0	10 336		
		SZGR2.0 (83)	1323		
	ncRNA	DisGeNET V7.0	120	662	
		SZDB2.0	463		
		SZGR2.0	6		
		RNADisease v4.0	146		
		CircR2Disease v2.0	5		
		LncRNADisease v3.0	2		
	Methylation	SZDB2.0	11 616	12 098	
		SZGR2.0	482		
Anxiety disorder	SNP	DisGeNET V7.0	169	169	999
	Gene	DisGeNET V7.0	795	795	
	ncRNA	DisGeNET V7.0	21	35	
		RNADisease v4.0	14		

Additionally, MDDOmics includes 10 839 entries of bipolar disorder, 27 369 entries of schizophrenia and 999 entries of anxiety disorder, with a substantial portion sourced from the DisGeNET (23) database. Moreover, for bipolar disorder, the primary data source was the dbBIP (29) database, whereas schizophrenia-related entries were primarily derived from the SZDB (27) and SZGR (28) databases.

### Search and analysis

To better accommodate users’ requirements, we designed a variety of detailed search ways according to different omics. Taking the SNP search as an example, users can not only search via the SNP ID but also leverage the gene name, disease phenotype and the range of SNP locations. Similarly, for metabolite searches, users have the flexibility to search using the Human Metabolome Database ID, PubChem ID, metabolite name, metabolite category and experiment tissue.

To ensure that users can access the required information more efficiently and conveniently, we have refined our search tools, incorporating several user-friendly features. For example, recognizing that some genes may possess multiple aliases, the database is configured to search simultaneously by both the gene names and their corresponding aliases. In addition, the database employs a fuzzy matching strategy for some complex attributes like protein and metabolite names, significantly improving the search flexibility.

In the differential analysis interface, we have curated four datasets as detailed in Table 2. These datasets encompass gene expression and methylation profile data derived from blood and brain tissue samples of MDD patients and their corresponding controls. Box plots with scatter overlay are employed to visualize the gene expression and methylation levels across samples in the collected GEO datasets. Additionally, the interface provides the results using differential analysis tools for users’ reference. It allows users to set custom thresholds and filter criteria, facilitating the rapid identification of differentially expressed genes, methylation sites and methylation regions of interest.

**Table 2.** Collected GEO datasets for differential analysis

Data type	GEO accession	Tissue	Participant count
Gene expression	GSE98793 (84)	Whole blood-batch 1	Case: 64 Control: 32
		Whole blood-batch 2	Case: 64 Control: 32
	GSE102556 (85)	Cingulate gyrus 25	Case: 13 Control: 15
		Orbitofrontal	Case: 26 Control: 22
		Anterior Insula	Case: 26 Control: 22
		Dorsolateral prefrontal cortex	Case: 26 Control: 22
		Nucleus accumbens	Case: 26 Control: 22
		Subiculum	Case: 24 Control: 19
Methylation	GSE201287 (86)	Blood	Case: 40 Control: 40
	GSE88890 (87)	Cortex, Brodmann area 11	Case: 20 Control: 20
		Cortex, Brodmann area 25	Case: 17 Control: 18

In the enrichment analysis interface, MDDOmics integrates the KEGG pathway and GO enrichment analysis results, derived from both conventional and WEAT (50) methods. Furthermore, the database presents the top 10 terms from these enrichment results in a bubble plot, aiding users in gaining a more intuitive grasp of potential MDD-related pathways, biological processes, cellular components and molecular functions. Notably, the outcomes of our enrichment analysis are largely consistent with existing findings. For instance, the ‘Neuroactive ligand-receptor interaction’ pathway, which consistently ranks at the forefront in all our KEGG pathway analyses, has been frequently implicated in association with MDD (68–71). In addition, the biological process term ‘superior temporal gyrus development’ ranks uniquely high in the enrichment analysis results using the gene essentiality score derived from blood expression profiles. This result is in alignment with many studies that have suggested the association between the volume of the superior temporal gyrus and MDD (72–75).

The prediction interface provides the users with selectable PPI networks and types of prediction targets, allowing them to obtain the prediction results for potential MDD-related genes, lncRNAs and miRNAs, excluding the significant MDD-related factors already in the database.

### Visualization

For the collected public data, MDDOmics offers three types of visualization interfaces for users to understand MDD more graphically and vividly.

The network from the ‘PPI’ interface illustrates the PPI interaction relationships of collected MDD-related genes. The nodes provide gene-related annotation information, while edges offer scores from the STRING (43) database, representing the degree of association between two genes in the network. Users can select the chromosomes of interest for their analyses. Additionally, by inputting a gene name, users can access and explore the corresponding sub-network in detail.

**Figure 2.**
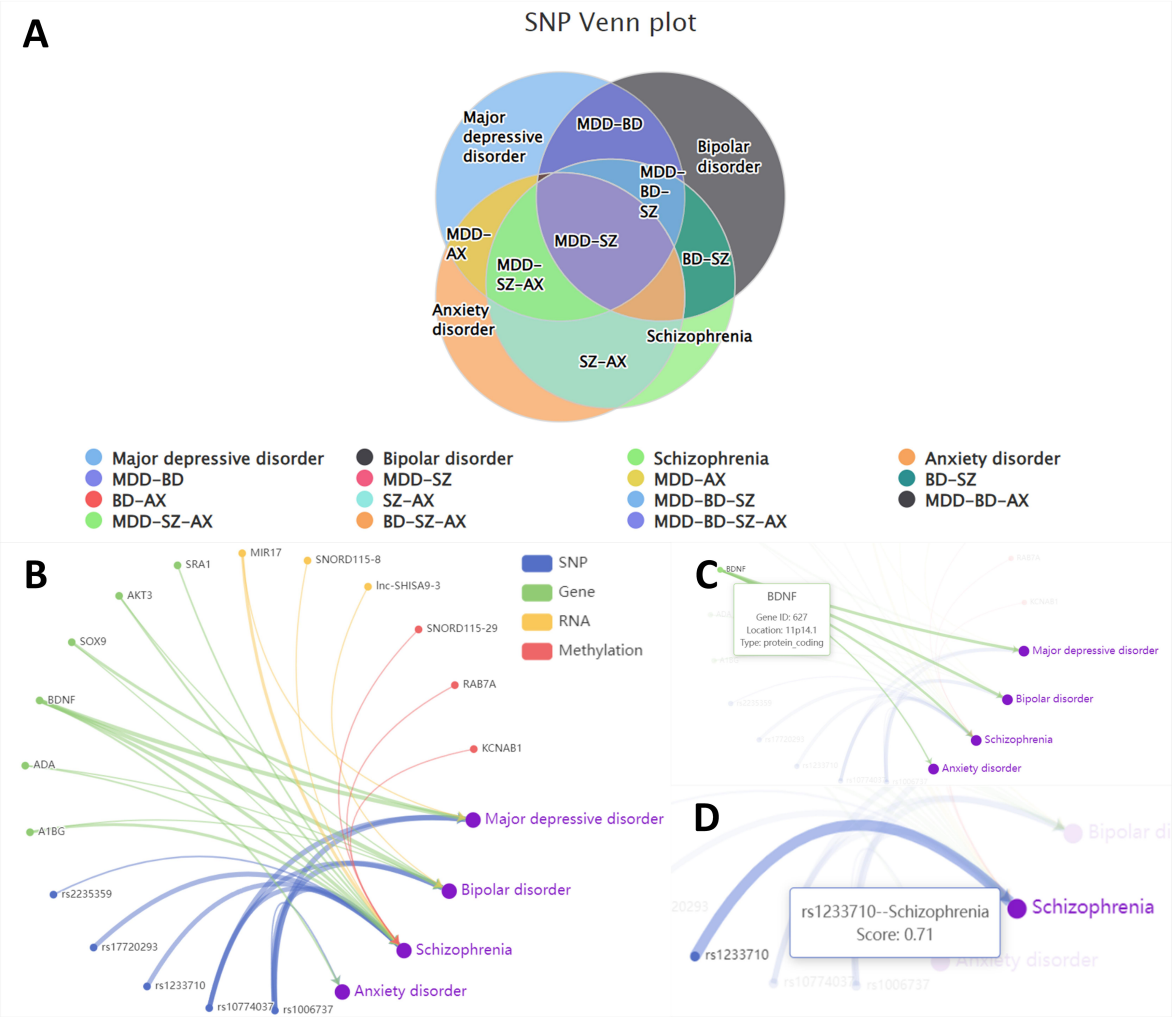
The ‘Relation’ interface shows the overlap of various omics data among different disorders by Venn plots (A). We also display the relationship between different entered omics data and disorders by the relationship network (B). Hover over nodes (C) and edges (D) to view specific information.

In the ‘Relation’ section, we show the similarities and differences of the biological factors among different disorders. From the disease perspective, we use a Venn diagram to show the intersection of the four disorders across four omics (Figure 2A). From the perspective of the omics data, we employ a relationship network to show associations between disorders and the biological factors provided by users (Figure 2B). In this network, nodes show the biological factor’s annotations (Figure 2C), while the edge thickness signifies the degree of disease-factor association as measured in DisGeNET (23) (Figure 2D).

**Figure 3.**
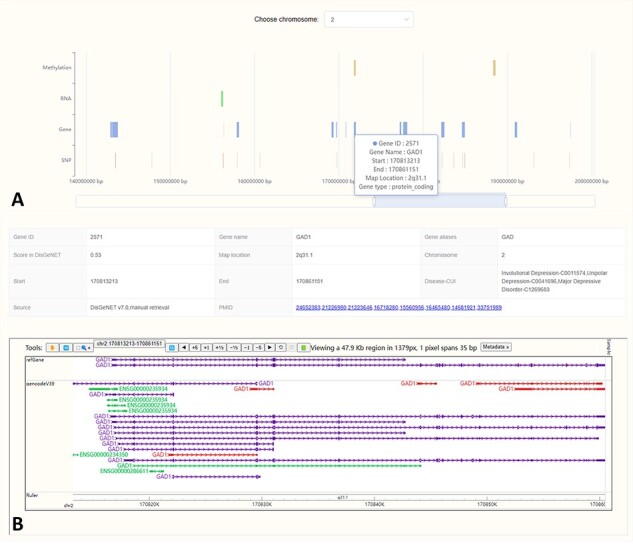
In the location visualization interface, we showed the locations of MDD-related biological factors on different chromosomes (A). Click the region of interest in the plot and the corresponding detailed annotations will be displayed below (B).

The ‘Location’ interface graphically presents the chromosomal locations of biological factors from four omics related to MDD. It enables users to swiftly compare the locations across different omics, facilitating the identification of regions for in-depth exploration (Figure 3A). By clicking on a biological factor in the visualization, users will be provided with detailed annotations from the MDDOmics database, as well as from the refGene and gencodeV39 tracks hosted by WashU (44) (Figure 3B).

## Conclusion

Considering the significant danger and widespread prevalence of MDD, there is an urgent need for a systematic platform to integrate multi-layered data from various sources. In response to this need, we developed MDDOmics, a comprehensive multi-omics database of MDD. The database integrates a large number of MDD-related entries and datasets, including SNPs, genes, ncRNAs, DNA methylations, metabolites and proteins. Several types of search and visualization interfaces are provided to make data easier to understand. It also integrates multi-omics data on schizophrenia, bipolar disorder and anxiety disorder, which are prone to clinical misdiagnosis with MDD. Several downstream analyses are conducted on the collected public data, including differential analysis, enrichment analysis and potential disease-gene predictions.

While this study has achieved progress in the field, it still has some limitations that provide important directions for future research. First, we attempted to ensure the accuracy and diversity of our assessments by retaining original correlation scores between biological factors and disorders from various sources. However, developing new scores based on a unified standard could give a more consistent perspective of data evaluations. Second, we currently rely on a limited amount of data retrieved manually from literature. Enhancing such collection efforts in the future will substantially expand the database’s comprehensiveness. Finally, our investigation into the correlations among different omics data was not deep enough. Future research should focus more on this for a more in-depth exploration and analysis to fully understand the mechanisms of MDD.

Overall, MDDOmics enables researchers to access rich, uniformly formatted multi-omics MDD data simultaneously and provides a feasible solution for the joint analysis of multi-omics data on MDD and MDD-similar disorders. It helps to develop subsequent MDD identification and stratification methods, providing researchers and clinicians with a clearer understanding of the intricate disease pathogenesis.

## Data Availability

All data relevant to this study are incorporated into the article or available online in the MDDOmics (https://www.csuligroup.com/MDDOmics).

## References

[1] American Psychiatric Association . (2013) *Diagnostic and Statistical Manual of Mental Disorders*, 5th edn. Arlington, VA, USA: American Psychiatric Publishing.

[2] World Health Organization . (2023) Depressive Disorder (Depression). https://www.who.int/news-room/fact-sheets/detail/depression (20 January 2024, date last accessed).

[3] Moussavi S. , Chatterji S., Verdes E. et al. (2007) Depression, chronic diseases, and decrements in health: results from the World Health Surveys. *Lancet*, 370, 851–858. 17826170 10.1016/S0140-6736(07)61415-9

[4] GBD 2016 Disease and Injury Incidence and Prevalence Collaborators . (2017) Global, regional, and national incidence, prevalence, and years lived with disability for 328 diseases and injuries for 195 countries, 1990–2016: a systematic analysis for the Global Burden of Disease Study 2016. *Lancet*, 390, 1211–1259. 28919117 10.1016/S0140-6736(17)32154-2 PMC5605509

[5] Mitchell A.J. , Vaze A. and Rao S. (2009) Clinical diagnosis of depression in primary care: a meta-analysis. *Lancet*, 374, 609–619. 19640579 10.1016/S0140-6736(09)60879-5

[6] Malhi G.S. and Mann J.J. (2018) Depression. *Lancet*, 392, 2299–2312. 30396512 10.1016/S0140-6736(18)31948-2

[7] Li Z ., Ruan M., Chen J. et al. (2021) Major depressive disorder: advances in neuroscience research and translational applications. *Neurosci. Bull*., 37, 863–880. 33582959 10.1007/s12264-021-00638-3 PMC8192601

[8] Otte C ., Gold S.M., Penninx B.W. et al. (2016) Major depressive disorder. *Nat. Rev. Dis. Primers*., 2, 16065. 10.1038/nrdp.2016.65 27629598

[9] Bierut L.J. , Heath A.C., Bucholz K.K. et al. (1999) Major depressive disorder in a community-based twin sample: are there different genetic and environmental contributions for men and women? *Arch. Gen. Psychiatry*, 56, 557–563. 10359473 10.1001/archpsyc.56.6.557

[10] Wray N.R. , Ripke S., Mattheisen M. et al. (2018) Genome-wide association analyses identify 44 risk variants and refine the genetic architecture of major depression. *Nat. Genet*., 50, 668–681. 29700475 10.1038/s41588-018-0090-3 PMC5934326

[11] Giannakopoulou O. , Lin K., Meng X. et al. (2021) The genetic architecture of depression in individuals of East Asian ancestry: a genome-wide association study. *JAMA Psychiatry*, 78, 1258–1269. 34586374 10.1001/jamapsychiatry.2021.2099 PMC8482304

[12] Leday G.G.R. , Vertes P.E., Richardson S. et al. (2018) Replicable and coupled changes in innate and adaptive immune gene expression in two case-control studies of blood microarrays in major depressive disorder. *Biol. Psychiatry*, 83, 70–80. 28688579 10.1016/j.biopsych.2017.01.021 PMC5720346

[13] Huls A. , Robins C., Conneely K.N. et al. (2020) Association between DNA methylation levels in brain tissue and late-life depression in community-based participants. *Transl. Psychiatry*, 10, 262. 10.1038/s41398-020-00948-6 PMC7393126 32733030

[14] Cordova-Palomera A. , Fatjo-Vilas M., Gasto C. et al. (2015) Genome-wide methylation study on depression: differential methylation and variable methylation in monozygotic twins. *Transl. Psychiatry*, 5, e557. 10.1038/tp.2015.49 PMC4462612 25918994

[15] Pu J. , Liu Y., Zhang H. et al. (2021) An integrated meta-analysis of peripheral blood metabolites and biological functions in major depressive disorder. *Mol. Psychiatry*, 26, 4265–4276. 31959849 10.1038/s41380-020-0645-4 PMC8550972

[16] Lin C.C. , Su H., Shiea J. et al. (2022) Isobaric tags for relative and absolute quantitation identification of blood proteins relevant to paroxetine response in patients with major depressive disorder. *Front. Psychiatry*, 13, 577857. 10.3389/fpsyt.2022.577857 PMC9058070 35509884

[17] Kennis M. , Gerritsen L., van Dalen M. et al. (2020) Prospective biomarkers of major depressive disorder: a systematic review and meta-analysis. *Mol. Psychiatry*, 25, 321–338. 31745238 10.1038/s41380-019-0585-z PMC6974432

[18] Hasin Y. , Seldin M. and Lusis A. (2017) Multi-omics approaches to disease. *Genome Biol*., 18, 83. 10.1186/s13059-017-1215-1 PMC5418815 28476144

[19] Karczewski K.J. and Snyder M.P. (2018) Integrative omics for health and disease. *Nat. Rev. Genet*., 19, 299–310. 29479082 10.1038/nrg.2018.4 PMC5990367

[20] Guo L. , Zhang W., Chang S. et al. (2012) MK4MDD: a multi-level knowledge base and analysis platform for major depressive disorder. *PLoS One*, 7, e46335. 10.1371/journal.pone.0046335 PMC3465288 23071556

[21] Pu J. , Yu Y., Liu Y. et al. (2020) MENDA: a comprehensive curated resource of metabolic characterization in depression. *Brief Bioinform*., 21, 1455–1464. 31157825 10.1093/bib/bbz055 PMC7373181

[22] Xiang J. , Zhang J., Zhao Y. et al. (2022) Biomedical data, computational methods and tools for evaluating disease-disease associations. *Brief Bioinform*., 23, bbac006. 10.1093/bib/bbac006 35136949

[23] Pinero J. , Ramirez-Anguita J.M., Sauch-Pitarch J. et al. (2020) The DisGeNET knowledge platform for disease genomics: 2019 update. *Nucleic Acids Res*., 48, D845–D855. 31680165 10.1093/nar/gkz1021 PMC7145631

[24] Chen J. , Lin J., Hu Y. et al. (2023) RNADisease v4.0: an updated resource of RNA-associated diseases, providing RNA-disease analysis, enrichment and prediction. *Nucleic Acids Res*., 51, D1397–D1404. 36134718 10.1093/nar/gkac814 PMC9825423

[25] Fan C. , Lei X., Tie J. et al. (2022) CircR2Disease v2.0: an updated web server for experimentally validated circRNA-disease associations and its application. *Genom. Proteom. Bioinform*., 20, 435–445. 10.1016/j.gpb.2021.10.002 PMC9801044 34856391

[26] Lin X. , Lu Y., Zhang C. et al. (2023) LncRNADisease v3.0: an updated database of long non-coding RNA-associated diseases. *Nucleic Acids Res*., 52, D1365–D1369. 10.1093/nar/gkad828 PMC10767967 37819033

[27] Wu Y. , Li X., Liu J. et al. (2020) SZDB2.0: an updated comprehensive resource for schizophrenia research. *Hum. Genet*., 139, 1285–1297. 32385526 10.1007/s00439-020-02171-1

[28] Jia P. , Han G., Zhao J. et al. (2017) SZGR 2.0: a one-stop shop of schizophrenia candidate genes. *Nucleic Acids Res*., 45, D915–D924. 27733502 10.1093/nar/gkw902 PMC5210619

[29] Li X. , Ma S., Yan W. et al. (2022) dbBIP: a comprehensive bipolar disorder database for genetic research. *Database*, 2022, baac049. 10.1093/database/baac049 PMC9250320 35779245

[30] Labonte B ., Engmann O., Purushothaman I. et al. (2017) Sex-specific transcriptional signatures in human depression. *Nat. Med*., 23, 1102–1111. 28825715 10.1038/nm.4386 PMC5734943

[31] Xiu J ., Li J., Liu Z. et al. (2022) Elevated BICD2 DNA methylation in blood of major depressive disorder patients and reduction of depressive-like behaviors in hippocampal Bicd2-knockdown mice. *Proc. Natl. Acad. Sci. U. S. A*., 119, e2201967119. 10.1073/pnas.2201967119 PMC9335189 35858435

[32] Murphy T.M. , Crawford B, Dempster E.L. et al. (2017) Methylomic profiling of cortex samples from completed suicide cases implicates a role for PSORS1C3 in major depression and suicide. *Transl. Psychiatry*, 7, e989. 10.1038/tp.2016.249 PMC5545719 28045465

[33] Edgar R. , Domrachev M. and Lash A.E. (2002) Gene Expression Omnibus: NCBI gene expression and hybridization array data repository. *Nucleic Acids Res*., 30, 207–210. 11752295 10.1093/nar/30.1.207 PMC99122

[34] Seal R.L. , Braschi B., Gray K. et al. (2023) Genenames.org: the HGNC resources in 2023. *Nucleic Acids Res*., 51, D1003–D1009. 36243972 10.1093/nar/gkac888 PMC9825485

[35] Maglott D. , Ostell J., Pruitt K.D. et al. (2011) Entrez Gene: gene-centered information at NCBI. *Nucleic Acids Res*., 39, D52–57. 21115458 10.1093/nar/gkq1237 PMC3013746

[36] Sherry S.T. , Ward M.H., Kholodov M. et al. (2001) dbSNP: the NCBI database of genetic variation. *Nucleic Acids Res*., 29, 308–311. 11125122 10.1093/nar/29.1.308 PMC29783

[37] Durinck S. , Spellman P.T., Birney E. et al. (2009) Mapping identifiers for the integration of genomic datasets with the R/Bioconductor package biomaRt. *Nat. Protoc*., 4, 1184–1191. 19617889 10.1038/nprot.2009.97 PMC3159387

[38] Wu T. , Hu E., Xu S. et al. (2021) clusterProfiler 4.0: a universal enrichment tool for interpreting omics data. *Innovation*, 2, 100141. 10.1016/j.xinn.2021.100141 PMC8454663 34557778

[39] Volders P.J. , Anckaert J., Verheggen K. et al. (2019) LNCipedia 5: towards a reference set of human long non-coding RNAs. *Nucleic Acids Res*., 47, D135–D139. 30371849 10.1093/nar/gky1031 PMC6323963

[40] Rophina M. , Sharma D., Poojary M. et al. (2020) Circad: a comprehensive manually curated resource of circular RNA associated with diseases. *Database*, 2020, baaa019. 10.1093/database/baaa019 PMC7100626 32219412

[41] Glazar P. , Papavasileiou P. and Rajewsky N. (2014) circBase: a database for circular RNAs. *RNA*, 20, 1666–1670. 25234927 10.1261/rna.043687.113 PMC4201819

[42] Kozomara A. , Birgaoanu M. and Griffiths-Jones S. (2019) miRBase: from microRNA sequences to function. *Nucleic Acids Res*., 47, D155–D162. 30423142 10.1093/nar/gky1141 PMC6323917

[43] Szklarczyk D. , Gable A.L., Lyon D. et al. (2019) STRING v11: protein-protein association networks with increased coverage, supporting functional discovery in genome-wide experimental datasets. *Nucleic Acids Res*., 47, D607–D613. 30476243 10.1093/nar/gky1131 PMC6323986

[44] Li D. , Purushotham D., Harrison J.K. et al. (2022) WashU Epigenome Browser update 2022. *Nucleic Acids Res*., 50, W774–W781. 35412637 10.1093/nar/gkac238 PMC9252771

[45] Kang J. , Tang Q., He J. et al. (2022) RNAInter v4.0: RNA interactome repository with redefined confidence scoring system and improved accessibility. *Nucleic Acids Res*., 50, D326–D332. 34718726 10.1093/nar/gkab997 PMC8728132

[46] Oughtred R. , Rust J., Chang C. et al. (2021) The BioGRID database: a comprehensive biomedical resource of curated protein, genetic, and chemical interactions. *Protein Sci*., 30, 187–200. 33070389 10.1002/pro.3978 PMC7737760

[47] Kim C.Y. , Baek S., Cha J. et al. (2022) HumanNet v3: an improved database of human gene networks for disease research. *Nucleic Acids Res*., 50, D632–D639. 34747468 10.1093/nar/gkab1048 PMC8728227

[48] Ziv M. , Gruber G., Sharon M. et al. (2022) The TissueNet v.3 database: protein-protein interactions in adult and embryonic human tissue contexts. *J. Mol. Biol*., 434, 167532. 10.1016/j.jmb.2022.167532 35662455

[49] Greene C.S. , Krishnan A., Wong A.K. et al. (2015) Understanding multicellular function and disease with human tissue-specific networks. *Nat. Genet*., 47, 569–576. 25915600 10.1038/ng.3259 PMC4828725

[50] Fan R. , Cui Q. and Vitek O. (2021) Toward comprehensive functional analysis of gene lists weighted by gene essentiality scores. *Bioinformatics*, 37, 4399–4404. 34170294 10.1093/bioinformatics/btab475

[51] Howard D.M. , Adams M.J., Clarke T.K. et al. (2019) Genome-wide meta-analysis of depression identifies 102 independent variants and highlights the importance of the prefrontal brain regions. *Nat. Neurosci*., 22, 343–352. 30718901 10.1038/s41593-018-0326-7 PMC6522363

[52] Major Depressive Disorder Working Group of the Psychiatric GWAS Consortium, Ripke S., Wray N.R. et al. (2013) A mega-analysis of genome-wide association studies for major depressive disorder. *Mol. Psychiatry*, 18, 497–511. 22472876 10.1038/mp.2012.21 PMC3837431

[53] Pain O. , Hodgson K., Trubetskoy V. et al. (2022) Identifying the common genetic basis of antidepressant response. *Biol. Psychiatry Glob. Open Sci*., 2, 115–126. 35712048 10.1016/j.bpsgos.2021.07.008 PMC9117153

[54] CONVERGE consortium, Bigdeli T.B. and Kretzschmar W. (2015) Sparse whole-genome sequencing identifies two loci for major depressive disorder. *Nature*, 523, 588–591. 26176920 10.1038/nature14659 PMC4522619

[55] Als T.D. , Kurki M.I., Grove J. et al. (2023) Depression pathophysiology, risk prediction of recurrence and comorbid psychiatric disorders using genome-wide analyses. *Nat. Med*., 29, 1832–1844. 37464041 10.1038/s41591-023-02352-1 PMC10839245

[56] Nagel M. , Jansen P.R., Stringer S. et al. (2018) Meta-analysis of genome-wide association studies for neuroticism in 449,484 individuals identifies novel genetic loci and pathways. *Nat. Genet*., 50, 920–927. 29942085 10.1038/s41588-018-0151-7

[57] Sud M. , Fahy E., Cotter D. et al. (2016) Metabolomics Workbench: an international repository for metabolomics data and metadata, metabolite standards, protocols, tutorials and training, and analysis tools. *Nucleic Acids Res*., 44, D463–470. 26467476 10.1093/nar/gkv1042 PMC4702780

[58] Vizcaino J.A. , Deutsch E.W., Wang R. et al. (2014) ProteomeXchange provides globally coordinated proteomics data submission and dissemination. *Nat. Biotechnol*., 32, 223–226. 24727771 10.1038/nbt.2839 PMC3986813

[59] Tian Y. , Morris T.J., Webster A.P. et al. (2017) ChAMP: updated methylation analysis pipeline for Illumina BeadChips. *Bioinformatics*, 33, 3982–3984. 28961746 10.1093/bioinformatics/btx513 PMC5860089

[60] Ritchie M.E. , Phipson B, Wu D. et al. (2015) limma powers differential expression analyses for RNA-sequencing and microarray studies. *Nucleic Acids Res*., 43, e47. 10.1093/nar/gkv007 PMC4402510 25605792

[61] Consortium G.T. (2013) The Genotype-Tissue Expression (GTEx) project. *Nat. Genet*., 45, 580–585. 23715323 10.1038/ng.2653 PMC4010069

[62] Gray J.P. , Muller V.I., Eickhoff S.B. et al. (2020) Multimodal abnormalities of brain structure and function in major depressive disorder: a meta-analysis of neuroimaging studies. *Am. J. Psychiatry*, 177, 422–434. 32098488 10.1176/appi.ajp.2019.19050560 PMC7294300

[63] Kupfer D.J. , Frank E. and Phillips M.L. (2012) Major depressive disorder: new clinical, neurobiological, and treatment perspectives. *Lancet*, 379, 1045–1055. 22189047 10.1016/S0140-6736(11)60602-8 PMC3397431

[64] Mora C. , Zonca V., Riva M.A. et al. (2018) Blood biomarkers and treatment response in major depression. *Expert Rev. Mol. Diagn*., 18, 513–529. 29701114 10.1080/14737159.2018.1470927

[65] Liu J.J. , Wei Y.B., Strawbridge R. et al. (2020) Peripheral cytokine levels and response to antidepressant treatment in depression: a systematic review and meta-analysis. *Mol. Psychiatry*, 25, 339–350. 31427752 10.1038/s41380-019-0474-5

[66] Xiang J. , Zhang J., Zheng R. et al. (2021) NIDM: network impulsive dynamics on multiplex biological network for disease-gene prediction. *Brief Bioinform*., 22, bbab080. 10.1093/bib/bbab080 33866352

[67] Zhou X ., Menche J., Barabasi A.L. et al. (2014) Human symptoms-disease network. *Nat. Commun*., 5, 4212. 10.1038/ncomms5212 24967666

[68] Li Y. , Miao P., Li F. et al. (2023) An association study of clock genes with major depressive disorder. *J. Affective Disord*., 341, 147–153. 10.1016/j.jad.2023.08.113 37633529

[69] Li Z. , Dang W., Hao T. et al. (2023) Shared genetics and causal relationships between major depressive disorder and COVID-19 related traits: a large-scale genome-wide cross-trait meta-analysis. *Front. Psychiatry*, 14, 1144697. 10.3389/fpsyt.2023.1144697 PMC10328439 37426090

[70] Han M. , Yuan L., Huang Y. et al. (2022) Integrated co-expression network analysis uncovers novel tissue-specific genes in major depressive disorder and bipolar disorder. *Front. Psychiatry*, 13, 980315. 10.3389/fpsyt.2022.980315 PMC9445988 36081461

[71] Zhang Y. , Li M., Wang Q. et al. (2020) A joint study of whole exome sequencing and structural MRI analysis in major depressive disorder. *Psychol. Med*., 50, 384–395. 30722798 10.1017/S0033291719000072

[72] Gong L. , He C., Yin Y. et al. (2017) Nonlinear modulation of interacting between COMT and depression on brain function. *Eur. Psychiatry*, 45, 6–13. 28728097 10.1016/j.eurpsy.2017.05.024

[73] Zackova L. , Jani M., Brazdil M. et al. (2021) Cognitive impairment and depression: meta-analysis of structural magnetic resonance imaging studies. *Neuroimage Clin*., 32, 102830. 10.1016/j.nicl.2021.102830 PMC8473769 34560530

[74] Gong J. , Wang J., Qiu S. et al. (2020) Common and distinct patterns of intrinsic brain activity alterations in major depression and bipolar disorder: voxel-based meta-analysis. *Transl. Psychiatry*, 10, 353. 10.1038/s41398-020-01036-5 PMC7573621 33077728

[75] McLellan Q. , Wilkes T.C., Swansburg R. et al. (2018) History of suicide attempt and right superior temporal gyrus volume in youth with treatment-resistant major depressive disorder. *J. Affective Disord*., 239, 291–294. 10.1016/j.jad.2018.07.030 30031248

[76] Pinero J., Ramirez-Anguita J.M., Sauch-Pitarch J. et al. (2020) The DisGeNET knowledge platform for disease genomics: 2019 update. *Nucleic Acids Res*., 48, D845–D855. 31680165 10.1093/nar/gkz1021 PMC7145631

[77] Chen J., Lin J., Hu Y. et al. (2023) RNADisease v4.0: an updated resource of RNA-associated diseases, providing RNA-disease analysis, enrichment and prediction. *Nucleic Acids Res*., 51, D1397–D1404. 36134718 10.1093/nar/gkac814 PMC9825423

[78] Fan C., Lei X., Tie J. et al. (2022) CircR2Disease v2.0: an updated web server for experimentally validated circRNA-disease associations and its application. *Genom. Proteom. Bioinform*., 20, 435–445. 10.1016/j.gpb.2021.10.002 PMC9801044 34856391

[79] Lin X., Lu Y., Zhang C. et al. (2023) LncRNADisease v3.0: an updated database of long non-coding RNA-associated diseases. *Nucleic Acids Res*., 52, D1365–D1369. 10.1093/nar/gkad828 PMC10767967 37819033

[80] Pu J., Yu Y., Liu Y. et al. (2020) MENDA: a comprehensive curated resource of metabolic characterization in depression. *Brief. Bioinform*., 21, 1455–1464. 31157825 10.1093/bib/bbz055 PMC7373181

[81] Li X., Ma S., Yan W. et al. (2022) dbBIP: a comprehensive bipolar disorder database for genetic research. *Database*, 2022, baac049. 10.1093/database/baac049 PMC9250320 35779245

[82] Wu Y., Li X., Liu J. et al. (2020) SZDB2.0: an updated comprehensive resource for schizophrenia research. *Hum. Genet*., 139, 1285–1297. 32385526 10.1007/s00439-020-02171-1

[83] Jia P., Han G., Zhao J. et al. (2017) SZGR 2.0: a one-stop shop of schizophrenia candidate genes. *Nucleic Acids Res*., 45, D915–D924. 27733502 10.1093/nar/gkw902 PMC5210619

[84] Leday G.G.R., Vertes P.E., Richardson S. et al. (2018) Replicable and coupled changes in innate and adaptive immune gene expression in two case-control studies of blood microarrays in major depressive disorder. *Biol. Psychiatry*, 83, 70–80. 28688579 10.1016/j.biopsych.2017.01.021 PMC5720346

[85] Labonte B., Engmann O., Purushothaman I. et al. (2017) Sex-specific transcriptional signatures in human depression. *Nat. Med*., 23, 1102–1111. 28825715 10.1038/nm.4386 PMC5734943

[86] Xiu J., Li J., Liu Z. et al. (2022) Elevated BICD2 DNA methylation in blood of major depressive disorder patients and reduction of depressive-like behaviors in hippocampal Bicd2-knockdown mice. *Proc. Natl. Acad. Sci. U. S. A*., 119, e2201967119. 10.1073/pnas.2201967119 PMC9335189 35858435

[87] Murphy T. M., Crawford B., Dempster E. L. et al. (2017) Methylomic profiling of cortex samples from completed suicide cases implicates a role for PSORS1C3 in major depression and suicide. *Transl. Psychiatry*, 7, e989. 10.1038/tp.2016.249 PMC5545719 28045465

